# Immune Modulation and the Development of Fowl Typhoid: A Model of Human Disease?

**DOI:** 10.3390/pathogens9100843

**Published:** 2020-10-15

**Authors:** Ying Tang, Michael Jones, Paul A. Barrow, Neil Foster

**Affiliations:** 1 School of Veterinary Medicine and Science, University of Nottingham, Nottingham LE12 5RD, UK; ying.tang.cn@outlook.com (Y.T.); Michael.jones@nottingham.ac.uk (M.J.); paul.barrow@nottingham.ac.uk (P.A.B.); 2 SRUC Aberdeen Campus, Craibstone Estate, Ferguson Building, Aberdeen AB21 9YA, UK

**Keywords:** typhoid, *S.* Gallinarum, chicken, human, immunomodulation

## Abstract

*Salmonella enterica* serovar Gallinarum (*S*. Gallinarum) is the cause of typhoid in chickens but the immune factors that may facilitate the development of typhoid have not been fully elucidated. We show that, in contrast to non-typhoid *S*. Enteritidis infection, *S*. Gallinarum significantly reduced nitrite ion production and expression of mRNA for heterophil granulocyte chemoattractants CXCLi2 and IL-6 in chicken monocyte-derived macrophages (chMDMs) (*p* < 0.05) at 6 h post-infection (pi). *S*. Gallinarum also reduced IFN-γ and IL-17 expression by CD4^+^ lymphocytes cultured with infected chMDMs for 5 days but did not induce a Th2 phenotype or anergy. In vivo, *S*. Gallinarum also induced significantly lower expression of CXCLi1, CXCLi2, IL-1β, IL-6 and iNOS mRNA in the caecal tonsil by day 2 pi (*p* < 0.05–0.01) and consistently lower levels of IFN-γ, IL-18, IL-12, and IL-17. In the spleen, *S*. Gallinarum induced significantly lower levels of iNOS and IFN-γ (*p* < 0.01 and 0.05 respectively) and consistently lower levels of IL-18 and IL-12 but significantly greater (*p* < 0.01) expression of anti-inflammatory IL-10 at day 4 and 5 pi when compared to *S*. Enteritidis. This immune phenotype was associated with transit from the intestinal tissues to the liver by *S*. Gallinarum, not observed following *S*. Enteritidis infection. In conclusion, we report an immune mechanism that may facilitate typhoid disease in *S*. Gallinarum-infected chickens. However, down-regulation of inflammatory mediators, upregulation of IL-10, and associated liver colonisation are also characteristic of human typhoid, suggesting that this may also be a useful model of typhoid in humans.

## 1. Introduction

A small number of the more than 2500 serotypes of *Salmonella enterica* subsp *enterica* characteristically produce typhoid disease in a restricted range of host species. These include: *Salmonella enterica* subsp *enterica* serovar Typhi in man, *S*. Gallinarum in fowl, *S.* Dublin in cattle, *S*. Choleraesuis in pigs, *S*. Abortusovis in sheep and *S*. Typhimurium in mice [1]. The pathogenesis of infection is similar in all cases with the main route of infection being the faecal/oral route with rapid transit from the intestine to the gall bladder, liver, and spleen before recycling back to the intestine where they are shed in faeces. In the case of *S*. Gallinarum, bacterial uptake by intestinal epithelial cells in vitro, is associated with down-regulation of pro-inflammatory cytokines, IL-1β and IL-6 [2,3,4], but comprehensive studies have not been reported which have investigated the possible immunological basis for the development of typhoid disease following *S*. Gallinarum infection, compared to non-typhoid infections with other *Salmonella* spp in chicken. e.g., *S*. Enteritidis.

Human typhoid is estimated to infect around 20 million people globally and is endemic in economically distressed regions with poor sanitation [5,6]. The vast majority of our knowledge regarding the immune response in human typhoid is accrued from the murine/*S*. Typhimurium model of disease or the use of the Ty21a vaccine, or experimentally attenuated *S*. Typhi strains, in human volunteers [7,8,9]. However, one study has reported that there are decreased levels of inflammatory mediators (IFN-γ and IL-17) in the serum of patients with acute typhoid compared to levels from convalescent patients [10]. This suggests that the development of human typhoid may occur due to immunomodulation by *S*. Typhi, which has also been implicated by in vitro studies using *S*. Gallinarum [2,3]. In humans, in vitro studies have shown that inhibition of the inflammatory response occurs due to expression of the Vi capsular antigen by *S*. Typhi. This includes reduced opsonisation, phagocytosis, and production of oxidative killing pathways [11] and IL-8 production via inhibition of Toll-like receptor signalling [12].

Vi antigen is not expressed by *S*. Typhimurium but one study has shown that insertion of *S*. Typhi Vi antigen into *S*. Typhimurium down-regulates inflammatory immune responses and promotes production of anti-inflammatory IL-10 in mice, which wild type *S*. Typhimurium were unable to do [13]. Thus, although *S*. Typhimurium infection in mice has provided a useful model of human typhoid, it is clear that it has some important limitations.

The aim of the study we report was therefore twofold. First, we set out to determine the immunological basis for the establishment of typhoid in chickens by comparing immune responses following infection with typhoid *S*. Gallinarum and non-typhoid *S*. Enteritidis. Secondly, we assessed the potential of *S*. Gallinarum infection in chickens as a relevant model of human typhoid.

## 2. Materials and Methods

### 2.1. S. enterica Strains

*S*. Enteritidis strain P125109 and *S.* Gallinarum strain 9 were cultured in nutrient broth (Oxoid, Basingstoke, UK) at 37 °C with shaking at 150 rpm prior to use in experimental infections either in vitro or *in vivo*. Their source, virulence, and infection characteristics have been described previously by this group [14,15,16].

### 2.2. In Vitro Survival of S. enterica in Isolated chMDMs

Commercial chicken peripheral whole blood collected from spent Lohmann Lite laying hens (Harlan Laboratories UK Ltd., Leicestershire, UK) was used to isolate chicken peripheral blood mononuclear cells (PBMCs) by density gradient centrifugation [17]. Conversion of PBMCs into chicken monocyte-derived macrophages (chMDMs) and chMDM enrichment was confirmed by flow cytometry analysis [17,18]. Isolated chMDMs were suspended in RPMI 1640 (Gibco, Life Technologies, Abingdon, UK) supplemented with fetal bovine serum (FBS) (10%, vol/vol) (Gibco, Life Technologies, UK), HEPES (20 mM) (Sigma-Aldrich, Gillingham, UK), gentamicin sulfate (50 μg/mL) (Sigma-Aldrich), streptomycin-penicillin (10 U/mL) (Gibco, Life Technologies), amphotericin B (Fungizone) (1.25 μg/mL) (Gibco, Life Technologies), and L-glutamine (2 mM) (Gibco, Life Technologies) and made up to 5 × 10^5^ cells/mL for the following studies.

In vitro invasion was performed by using a multiplicity of infection (MOI) of 10. After 1 h of incubation of *S.* Enteritidis or *S*. Gallinarum in RPMI 1640 media chMDMs, the medium was replaced with fresh culture medium containing 100 μg/mL of gentamicin sulfate, and the cell preparation was incubated for another hour to kill extracellular bacteria. *Salmonella*-infected chMDMs were then kept in fresh culture medium supplemented with 20 μg/mL of gentamicin sulfate in subsequent studies. *S*. Enteritidis lipopolysaccharide (LPS) (50 μg/mL) (Sigma-Aldrich) was used as a positive control for nitrite ion (NO_2_^−^) and cytokine production, and phosphate-buffered saline (PBS) only was used as a negative control. At 2, 6, 24 and 48 h after infection, chMDMs infected with *Salmonella* were lysed by using Triton X-100 (1%, vol/vol) (Thermo Fisher Scientific, Abingdon, UK) to release and determine the intracellular survival of bacteria (log_10_ CFU/mL). The concentration of NO_2_^−^ produced by infected or uninfected cells was assessed by a Griess assay kit (Promega, Fitchburg, WI, USA). Cytokine mRNA expression levels were analysed by quantitative real-time PCR (qRT-PCR).

All experiments were carried out in triplicate using blood from three separate chickens.

### 2.3. Isolation of Chicken CD4^+^ T Cells and Avian Macrophage/CD4^+^ T Cell Model In Vitro

Chicken CD4^+^ T cells were positively-selected from peripheral whole blood using mouse-anti-chicken CD4 monoclonal antibody (MAb) (clone CT-4; Southern Biotech, Fitchburg, WI, USA) and anti-mouse IgG1 microbeads (Miltenyi Biotec, Woking, UK) by magnetic activated cell sorting (MACS) according to the manufacturers’ instructions. Chicken CD4^+^ T cells were then co-cultured with chMDMs and infected with either *S*. Enteritidis or *S*. Gallinarum at a ratio of 10:1 (chMDMs to CD4^+^ T cells) for 5 days [18]. Moreover, three control groups were set up as described previously. Briefly, (1) negative controls for viability and non-specific proliferation of CD4^+^ T cells (CD4^+^ T cells cultured alone), (2) controls for the allogeneic immune response (co-culture of uninfected chMDMs with CD4^+^ T cells), (3) positive controls for the in vitro proliferation of CD4^+^ T cells (CD4^+^ T cells cultured with concanavalin A (ConA) (10 μg/mL) (Sigma-Aldrich) as reported previously [18]. After 5 days of co-culture, CD4^+^ T cells from each group were collected to measure cytokine mRNA expression by qRT-PCR and proliferation of CD4^+^ T cells using the CellTiter96AQ_ueous_ One Solution cell proliferation assay (Promega). CD4^+^ T cells were also harvested from each group after 5 days of co-culture to measure cytokine mRNA expression by qRT-PCR.

As indicated above, all experiments were carried out in triplicate using blood from three separate chickens.

### 2.4. Phenotypic Analysis of Infected chMDMs and CD4^+^T Cells from Co-Culture with chMDMs

**Figure 1:**
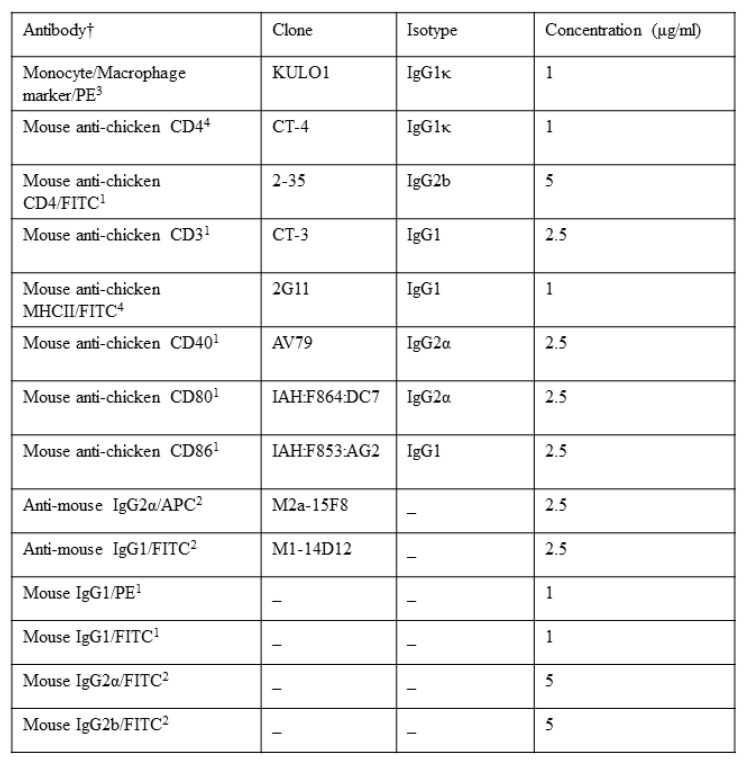
Monoclonal antibodies used to determine cell phenotype and activation. † Suppliers: 1, AbD Serotec, Kidlington, UK; 2, eBioscience, Abingdon, UK; 3, Santa Cruz Biotechnology, Santa Cruz, CA, USA; 4, Southern Biotech, Birmingham, Al, USA.

Cells collected for MHCII, CD40, CD80, CD86 (chMDMs) or CD28 expression were fixed with PBS containing formaldehyde (4% vol/vol) prior to incubation with the antibodies indicated and their isotype controls (shown in Figure 1). Fluorescence analysis was performed by using a FACSCanto II equipped with FACSDiva software (BD Biosciences, Wokingham, UK).

### 2.5. Salmonella Infection In Vivo

Two-day-old Lohmann Lite chickens were purchased from the Millennium Hatchery (Birmingham, UK). Two groups, each with 12 birds, were inoculated orally with 10^8^ CFU of *S*. Enteritidis or *S*. Gallinarum in 0.1 mL of nutrient broth. Another 12 birds inoculated orally with 0.1 mL of PBS were in the control group. Three groups in separate pens were given access to antibiotic-free feed and water *ad libitum* throughout the experiment. At 1, 2, 4, and 5 days post-inoculation, three birds from each group were sacrificed. Caecal contents and liver were collected aseptically and processed to determine bacterial counts using Brilliant Green agar plates containing sodium nalidixate (20 μg/mL; Sigma-Aldrich) and novobiocin (1 μg/mL; Sigma-Aldrich). Spleen and cecal tonsils were collected for cytokine mRNA expression analysis [18]. All animal care and experimentation were carried out under Home Office project license PPL 40/3412 and had local ethical approval from the University of Nottingham Animal Welfare and Ethical Review Body, April 2015.

### 2.6. Gene Expression Analysis by qRT-PCR

**Figure 2:**
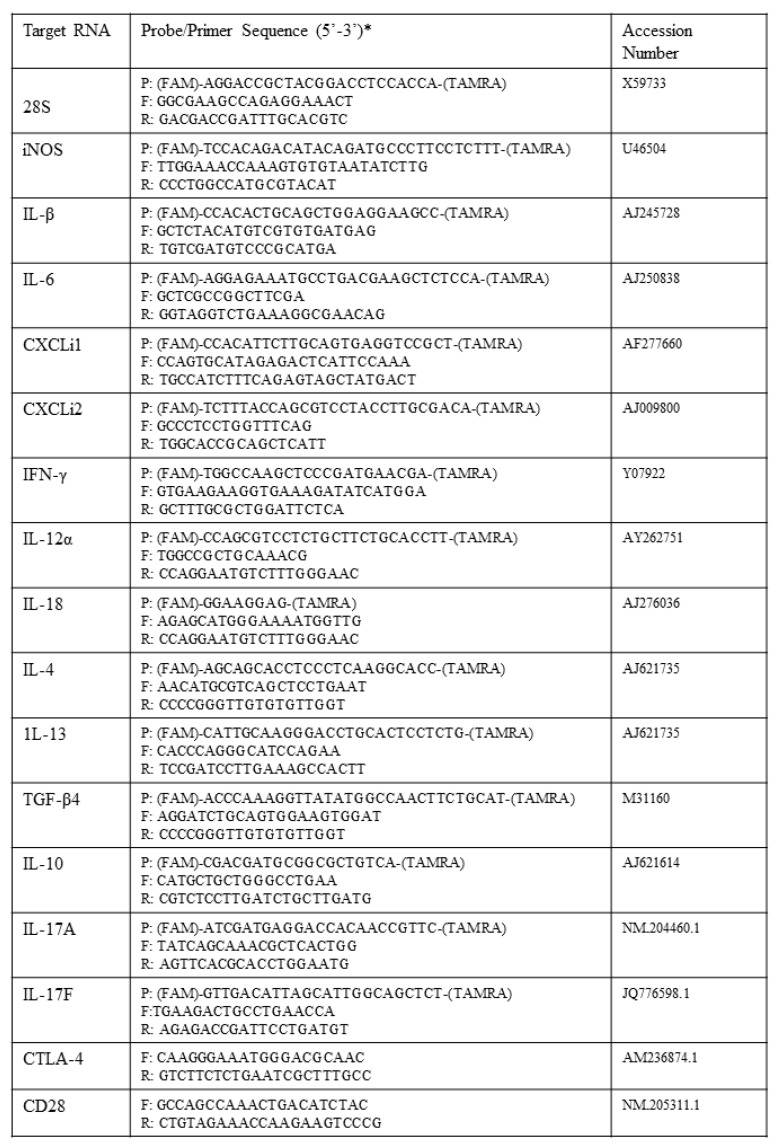
qPCR primer and probe sequences used in this study. * P = Probe; F = Forward primer; R = Reverse primer; FAM = 6-carboxyfluorescein; TAMRA = 6-carboxytetramethylrhodamine.

Total RNA was prepared using the RNeasy Plus minikit (Qiagen, Manchester, UK) prior to reverse transcription into cDNA by using a Transcriptor first-strand cDNA synthesis kit (Roche, Welwyn Garden City, UK) following the manufacturer’s instructions. Quantification of the gene expression levels of selected cytokines and chemokines was based on the LightCycler 480 system (Roche). All primer and probe sequences used in the study are shown in Figure 2. Gene expression of CD28 and CTLA-4 was detected by SYBR green-based qRT-PCR. To account for variation in sample preparation, the Ct values for target gene product for each sample were normalised using the Ct value of reference gene (28S) product for the same sample. Standard plots of Ct against log_10_(cDNA) of five-point five-fold serial dilutions were obtained for either reference gene (S′) or each target gene (S). Normalised Ct values were calculated using the formula Ct + (Nt − Ct′) × S/S′, with Nt as the mean Ct for 28S RNA among all samples, Ct′ the mean Ct for 28S RNA in the sample and S and S′ the slopes of the regressions of the standard plots for the cytokine mRNA and the 28S RNA, respectively. This effectively achieves interpolations on the standard plots to obtain the cytokine Ct values that would have been obtained had all samples had the same (mean) amount of 28S RNA. Results are expressed as fold difference from levels in controls using the formula: F = 2(T − C), with T as the mean corrected 40-Ct for test sample and C the mean corrected 40-Ct for control sample [2,19].

### 2.7. Statistical Analysis

Data were plotted and analysed by using GraphPad Prism 6.0 (GraphPad Software, San Diego, CA, USA). Comparisons between different groups and between different groups at different time points were performed by using two-way analysis of variance (ANOVA) followed by Tukey’s multiple comparison post hoc test. Statistical significance was determined at the 5% and 1% confidence limits as *p* values of <0.05 and <0.01.

## 3. Results

### 3.1. S. Gallinarum and S. Enteritidis Show Differential Infection and Survival Patterns in chMDMs

**Figure 3:**
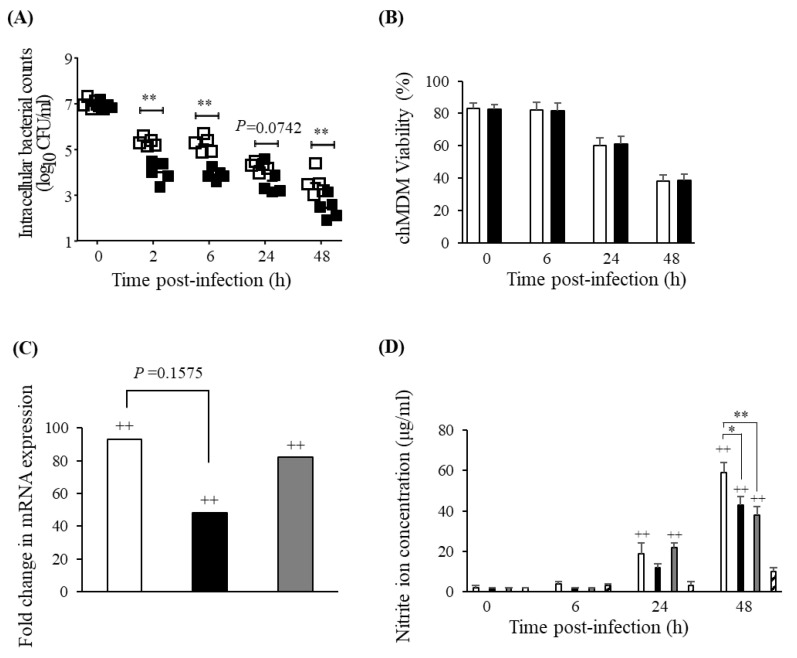
Survival dynamics and induction of reactive nitrogen species by chMDMs infected with *S*. Gallinarum or *S*. Enteritidis. Mean survival of *S*. Enteritidis (white bars) and *S*. Gallinarum (black bars) in chMDMs is shown in (**A**) and the effect of bacterial infection on mean chMDM viability is shown in (**B**) over a 48 h post-infection period. (**C**) Mean iNOS mRNA expression in chMDMs infected with *S*. Enteritidis (white bar), *S*. Gallinarum (black bar) or cultured with LPS (grey bar) after 6 h post-infection is shown as fold changes above iNOS mRNA expression in uninfected control chMDMs, given an arbitrary value of 1. (**D**) Mean nitrite ion (NO_2_^−^) concentration in supernatants isolated from cultures of chMDMs infected with *S*. Enteritidis (white bar), *S*. Gallinarum (black bar), cultured with LPS (grey bar) or in cultures from uninfected control chMDMs (diagonal stripe bar) over a 48 h post-infection/culture period. All means were calculated from results obtained from duplicate cultures of chMDMs obtained from 3 individual chickens. Data in panel (**B**) and (**D**) are presented as mean ± SEM (*n* = 3). (+) indicates statistically significant difference from negative control (++ *p* < 0.01); (*) indicates statistical differences between different treatments (* *p* < 0.05, ** *p* < 0.01).

*S*. Gallinarum infected and were phagocytosed by chMDM at significantly lower levels compared to *S*. Enteritidis during the initial 2 h post-infection (pi) period (*p* < 0.01) and this was followed by a lower rate of decline in the number of *S*. Gallinarum isolated from chMDM compared to *S*. Enteritidis after 48h pi (Figure 3A). The viability of infected chMDMs was maintained at over 80% until 6 h pi but was reduced to around 60% 24 h pi and 25% by 48 h pi but the effect of *S*. Gallinarum or *S*. Enteritidis on chMDM viability was comparable across all time points measured (Figure 3B). After 6 h pi, expression of iNOS mRNA was significantly upregulated in chMDMs at 6 h pi with either *S*. Gallinarum or *S*. Enteritidis (*p* < 0.01), and although the level of iNOS expression was not significantly different between *S*. Gallinarum or *S*. Enteritidis-infected chMDMs, *S*. Enteritidis induced on average around a 40-fold increase above that measured in *S*. Gallinarum-infected cells (Figure 3C). This trend was also reflected down-stream by relative NO_2_^−^ concentrations. Although neither serovar induced significantly increased levels before 24 h pi, at 24 and 48 h pi *S*. Enteritidis induced significantly increased NO_2_^−^ concentrations above uninfected controls (*p* < 0.01) and significantly greater concentrations compared to *S*. Gallinarum-infected chMDMs at 48 h pi (*p* < 0.05). In comparison, *S*. Gallinarum induced significantly higher NO_2_^−^ concentrations compared to uninfected controls only at 48h pi (*p* < 0.01) but even at 24 h pi there was a 10-fold increase, but this was not statistically significant (*p* > 0.05) (Figure 3D).

### 3.2. S. Gallinarum Induces Lower Expression of Inflammatory Cytokines and Chemokines by chMDMs Compared to S. Enteritidis

**Figure 4:**
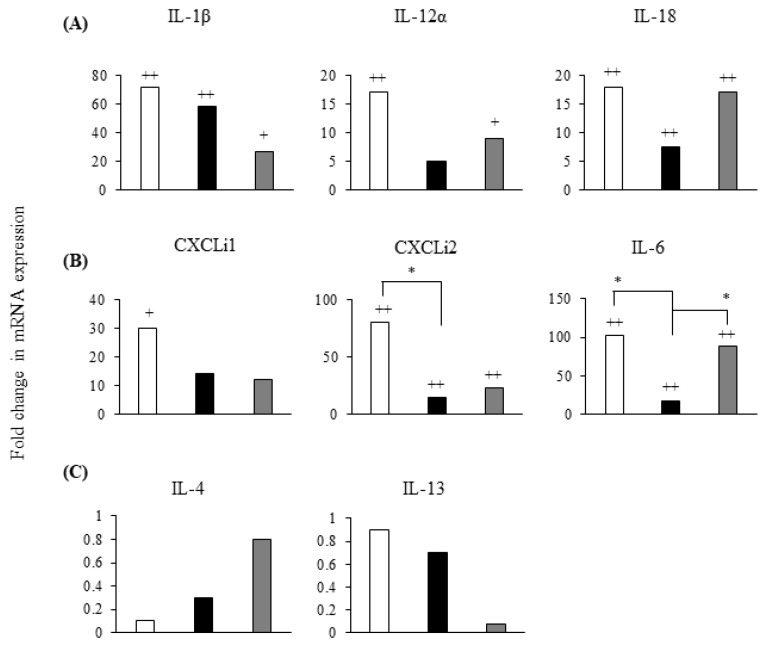
*S*. Gallinarum infection induces lower expression of inflammatory mediators than *S*. Enteritidis by chMDMs. Mean expression of pro-inflammatory cytokines mRNA (**A**); chemokines mRNA (**B**) and anti-inflammatory cytokines mRNA (**C**) is shown in chMDMs infected for 6 h with *S*. Enteritidis (white bars), *S*. Gallinarum (black bars) or cultured for 6 h with LPS (grey bars). Means were determined from duplicate chMDM cultures derived from 3 chickens (3 independent experiments with duplicate internal controls) and compared as fold changes to mRNA expression measured in uninfected controls (given an arbitrary value of 1). (+) indicates differences between levels of cytokines induced by each serovar compared to PBS-treated uninfected control, + *p* < 0.05, ++ *p* < 0.01; (*) indicates differences between levels of cytokines induced by different serovars, * *p* < 0.05.

At 6 h pi, *S*. Gallinarum induced much lower levels of mRNA expression for inflammatory cytokines IL-1β, IL-12α, and IL-18 when compared to *S*. Enteritidis-infected chMDMs (Figure 4A). *S*. Gallinarum also induced much lower levels of mRNA expression for chemokines (Figure 4B), and these differences were statistically significant for CXCLi2 and IL-6 (*p* < 0.05) (Figure 4B). Neither serovar induced differential expression of IL-4 or IL-13 mRNA (*p* > 0.05) (Figure 4C).

### 3.3. Different Strains of S. Gallinarum and S. Enteritidis Induced Similar Cytokine mRNA Expression Profiles in chMDMs

**Figure 5:**
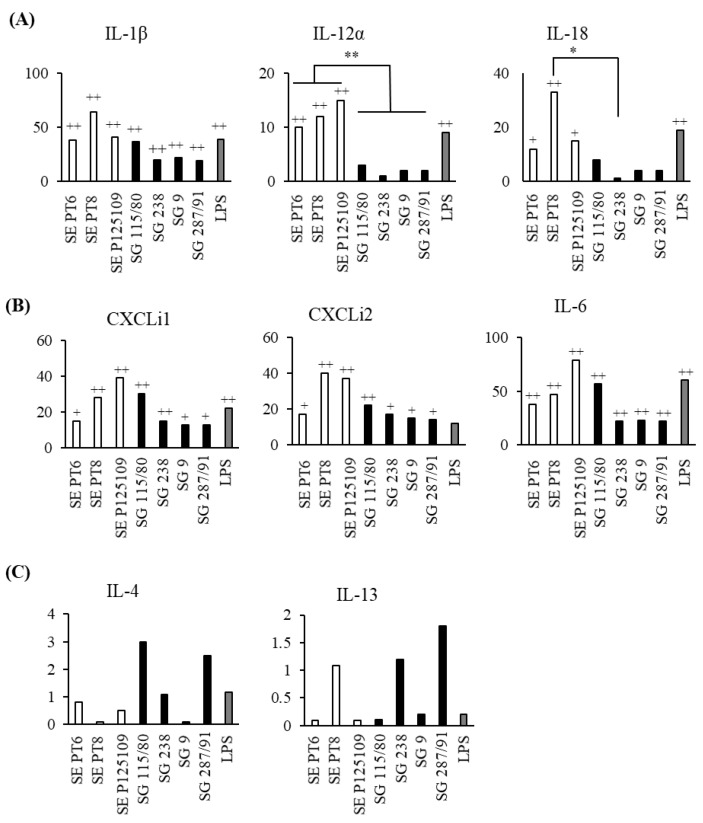
Trends in mRNA expression of inflammatory mediators are maintained in chMDMs using different strains of *S*. Gallinarum and *S*. Enteritidis. Mean expression of pro-inflammatory cytokines mRNA (**A**); chemokines mRNA(**B**) and anti-inflammatory cytokines (**C**) mRNA is shown in chMDMs infected for 6 h with different strains of *S*. Enteritidis (SE) (white bars), *S*. Gallinarum (SG) (black bars) or cultured for 6 h with LPS (grey bars). Means were determined from duplicate chMDM cultures derived from 3 chickens (3 independent experiments with duplicate internal controls) and compared as fold changes to mRNA expression measured in uninfected controls (given an arbitrary value of 1). (+) indicates differences between levels of cytokines induced by each serovar compared to uninfected control, + *p* < 0.05, ++ *p* < 0.01; (*) indicates differences between levels of cytokines induced by different serovars, * *p* < 0.05, ** *p* < 0.01.

To assess whether the trends in cytokine production by infected chMDMs were representative of *S*. Gallinarum and *S*. Enteritidis more generally, we repeated the cytokine study using different strains of the two serovars. Overall, the trends in cytokine expression by chMDMs infected with different strains of the two serovars were similar to that shown in Figure 5, with *S*. Enteritidis strains, inducing greater expression of proinflammatory cytokine mRNAs compared to *S*. Gallinarum strains, IL-12α (*p* < 0.01) and IL-18 (*p* < 0.05) were expressed significantly higher (Figure 5A). Similarly, chemokine expression was higher in chMDMs infected with *S*. Enteritidis compared to *S*. Gallinarum (Figure 5B). However, two *S*. Gallinarum strains (115/80 and 287/91) induced a greater than 2-fold increase in IL-4 mRNA expression by chMDMs compared to uninfected controls, although these differences were not statistically significant (*p* > 0.05) (Figure 5C).

### 3.4. S. Enteritidis Induces a Dominant Th17/IFN-γ Response in CD4^+^ Lymphocytes Compared to S. Gallinarum

**Figure 6:**
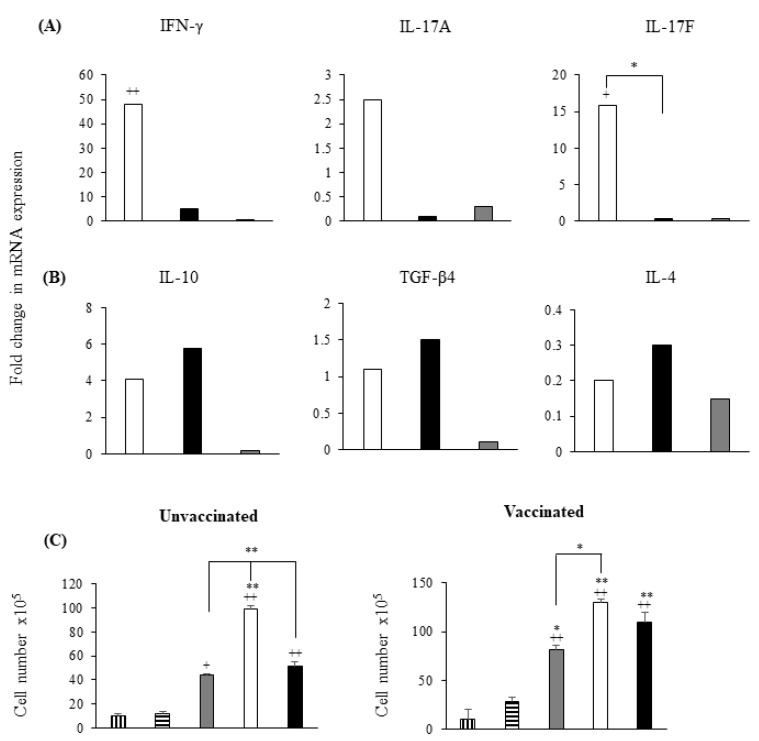
*S*. Gallinarum-infected chMDMs fail to induce differential expression of IFNγ/IL-17 responses by CD4^+^ lymphocytes. Data plots (shown in panels (**A**,**B**)) show mean mRNA expression of pro-inflammatory (**A**) and anti-inflammatory (**B**) cytokines by CD4^+^ lymphocytes cultures for 5 days with chMDMs infected with *S*. Enteritidis (white bars); *S*. Gallinarum (black bars) or LPS (grey bars). Means were determined from duplicate chMDM cultures derived from 3 chickens (3 independent experiments with duplicate internal controls) and cytokine expression is shown as a fold change above control levels (expression in CD4^+^ lymphocytes cultured with uninfected chMDMs for 5 days) given an arbitrary value of 1. Data plots shown in panel (**C**) = CD4^+^ lymphocyte proliferation after 5 days culture with chMDMs infected with *S*. Enteritidis (white bars); *S*. Gallinarum (black bars); stimulated with ConA (grey bars); CD4^+^ lymphocytes cultured with uninfected chMDMs (horizontal bars) or CD4^+^ lymphocytes cultured only in media for the 5 day experimental period (vertical bars). (+) indicates differences between levels of cytokines induced by each serovar compared to uninfected control, + *p* < 0.05, ++ *p* < 0.01; (*) indicates differences between levels of cytokines induced by different serovars, * *p* < 0.05, ** *p* < 0.01.

To determine whether *S*. Enteritidis-infected chMDMs induced a different CD4^+^ profile compared to *S*. Gallinarum, we co-cultured infected chMDMs with CD4^+^ lymphocytes for 5 days. After 5 days pi, expression of IFN-γ, IL-17a, and IL-17F mRNA was much greater in CD4^+^ lymphocytes cultured with *S*. Enteritidis-infected chMDMs than *S*. Gallinarum-infected chMDMs. However, although IFN-γ and IL-17a expression were increased by >40 and 2-fold respectively, only IL-17F expression was statistically greater (*p* < 0.05) (Figure 6A). TGF-β4, IL-4, and IL-10 were not differentially expressed by CD4^+^ lymphocytes cultured with either *S*. Enteritidis or *S*. Gallinarum-infected chMDMs compared to controls (Figure 6B). However, although IL-10 expression was increased 4-fold by *S*. Enteritidis and almost 6-fold by *S*. Gallinarum, these differences were not statistically significant (*p* > 0.05) (Figure 6B).

Proliferation of CD4^+^ lymphocytes from unvaccinated layer/breeder chickens was significantly increased (*p* < 0.01) when cultured for 5 days with *S*. Enteritidis-infected chMDMs compared to proliferation of CD4^+^ lymphocytes cultured with *S*. Gallinarum-infected chMDMs, CD4^+^ lymphocytes stimulated with ConA or unstimulated allogenic controls, over the same time period (Figure 6C). However, *S*. Gallinarum-infected chMDMs did still increase CD4^+^ proliferation above that measured in ConA-stimulated populations and a significantly higher (*p* < 0.01) proliferation when compared to allogeneic controls (Figure 6C). A similar trend was seen using CD4^+^ lymphocytes from vaccinated chickens but the level of proliferation of CD4^+^ lymphocytes in all experimental groups was higher when compared to unvaccinated chickens (Figure 6C).

### 3.5. S. Gallinarum Stimulates Lower Levels of Co-Stimulatory Molecules on the Surface of chMDMs Compared to S. Enteritidis but a Similar Expression Profile for CD28 on CD4^+^ Lymphocytes

**Figure 7:**
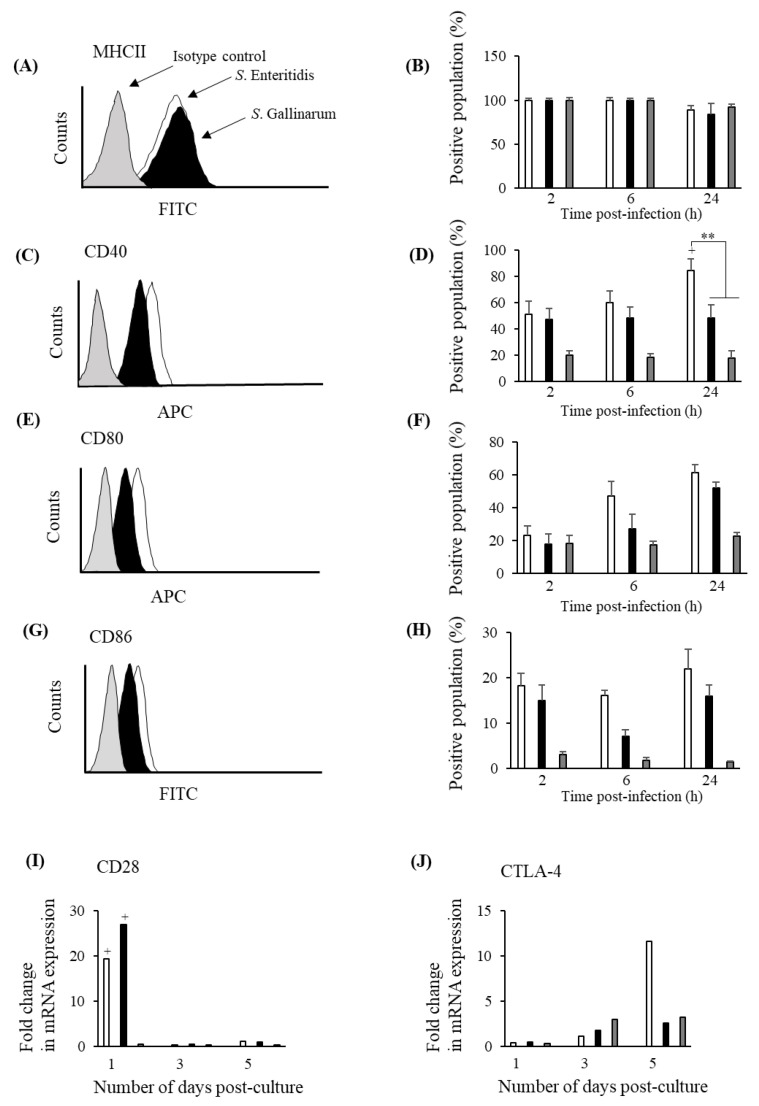
*S*. Gallinarum and *S*. Enteritidis infections induce similar expression of co-stimulatory molecules on the cell membranes of chMDMs and mRNA expression of CD28 and CTLA-4 in CD4^+^ lymphocytes FACS plots (shown in panels (**A**,**C**,**E**,**G**)) show representative expression of co-stimulatory molecules on the surface of chMDMs after 24 h post-infection. Expression levels are shown the surface of isotype controls (grey); chMDMs infected with *S*. Enteritidis (white) or *S*. Gallinarum (black). The percentage of positive chMDMs expressing co-stimulatory molecules over a 24 h post-infection period with *S*. Enteritidis (white bars); *S*. Gallinarum (black bars) or cultured with LPS (grey bars) are shown in panels (**B**,**D**,**F**,**H**). Expression of CD28 and CTLA-4 mRNA in CD4^+^ lymphocytes cultured over a 5 day period with chMDMs infected with *S*. Enteritidis (white bars); *S*. Gallinarum (black bars) or cultured with LPS (grey bars) are shown in (**I**) and (**J**) respectively. Mean values (shown in (**A**,**D**,**F**,**H**,**I**,**J**)) were determined from duplicate chMDM cultures derived from 3 chickens (3 independent experiments with duplicate internal controls) and CD28/CTLA-4 expression (shown in panels (**I**) and (**J)** respectively) is shown as a fold change above control levels (expression in CD4^+^ lymphocytes cultured with uninfected chMDMs for 5 days) given an arbitrary value of 1. Data in panel (**B**,**D**,**F**,**H**) are presented as mean ± SEM (*n* = 3).

Similar levels of MHCII expression were detected on the surface of chMDMs infected with either *S*. Gallinarum or *S*. Enteritidis at 24 h pi (Figure 7A) and in both cases the percentage of cells expressing MHCII was not significantly different to expression in uninfected controls (*p* > 0.05) (Figure 7B). Expression of co-stimulatory molecules (CD40, CD80 and CD86) on the surface of chMDMs was higher in *S*. Enteritidis-infected chMDMs compared to *S*. Gallinarum-infected chMDMs at all time points 2–24 h pi (Figure 7C–H). However, the percentage of chMDMs expressing CD40 was significantly greater (*p* < 0.01) following *S*. Enteritidis infection compared to *S*. Gallinarum at 24 h pi (Figure 7D). CD28 CTLA-4 and mRNA expression was also measured in CD4^+^ lymphocytes cultured with *S*. Enteritidis or *S*. Gallinarum-infected chMDMs for 5 days. CD28 mRNA expression was significantly increased (*p* < 0.05) in CD4^+^ lymphocytes at only at d1 post-culture with either *S*. Enteritidis or *S*. Gallinarum-infected chMDMs compared to allogeneic, uninfected controls but CD28 expression was not significantly different when comparing the effect of the two serovars (*p* > 0.05) (Figure 7I). CTLA-4 mRNA expression was increased around 10-fold (*S*. Enteritidis infection) and 3-fold (*S*. Gallinarum infection) at d5 post-culture, but these were not statistically significant (*p* > 0.05) (Figure 7I).

### 3.6. S. Gallinarum Effectively Disseminates from the Gastro-Intestinal Tract to Colonise Deeper Tissues

**Figure 8:**
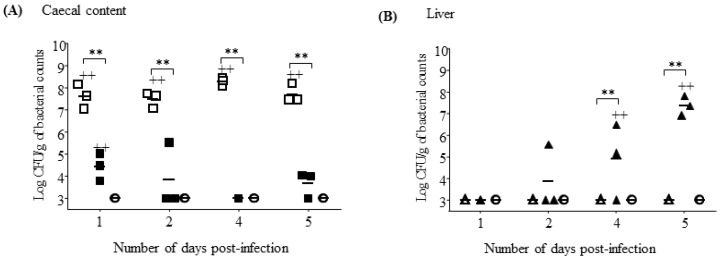
*S*. Gallinarum efficiently transits from the caeca to liver during a 5 day post-infection period *in vivo* Data plot (**A**) shows the mean numbers of *S*. Enteritidis (white squares) and *S*. Gallinarum (black squares) isolated from the caecal content of chickens over a 5 day post-infection period. Data plot (**B**) shows the mean numbers of *S*. Enteritidis (white triangles) and *S*. Gallinarum (black triangles) isolated from the liver of chickens over a 5 day post-infection period. Uninfected controls are shown by white circles. Each symbol represents data for an individual chicken, mean values were calculated from three chickens in each group in one independent experiment. (+) indicates differences between levels of cytokines induced by each serovar compared to uninfected control, ++ *p* < 0.01; (*) indicates differences between levels of cytokines induced by different serovars, ** *p* < 0.01.

The number of *S*. Enteritidis cultured from the caecum remained high (>1 × 10^7^) from d1–d5 pi, and on all days pi this was significantly greater than the numbers of *S*. Gallinarum recovered (*p* < 0.01) (Figure 8A). In contrast, the numbers of *S*. Gallinarum cultured from liver tissues steadily increased from d1–d5 pi and at d4 and d5 these were significantly greater than the numbers of *S*. Enteritidis recovered (*p* < 0.05) (1 × 10^7^ compared to 1 × 10^3^ at day 5), which remained consistently very low over the experimental period (Figure 8B).

### 3.7. S. Gallinarum Suppresses the Chemokine Response in the Caecal Tonsil In Vivo

**Figure 9:**
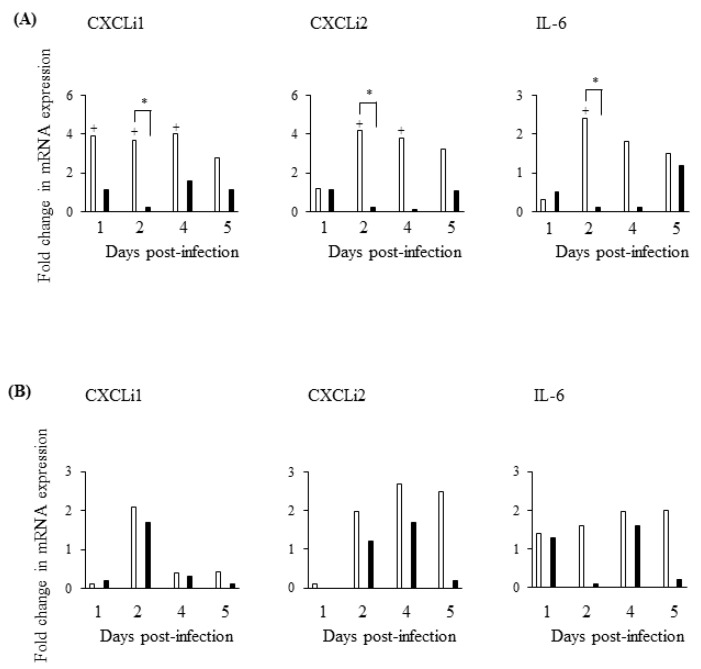
*S*. Gallinarum suppresses upregulation of chemokines in the caecal tonsils compared with *S*. Enteritidis infection in vivo Mean expression of chemokine mRNA is shown in the caecal tonsils (**A**) and spleens (**B**) of chickens infected with *S*. Enteritidis (white bars) or *S*. Gallinarum (black bars) over a 5 day post-infection period. Means were determined from data obtained from 3 independent experiments (individual birds) and compared as fold changes to mRNA expression measured in uninfected controls (given an arbitrary value of 1). (+) indicates differences between levels of cytokines induced by each serovar compared to PBS-treated uninfected control, + *p* < 0.05; (*) indicates differences between levels of cytokines induced by different serovars, * *p* < 0.05.

Following *S*. Gallinarum infection, chemokine (CXCLi1, CXCLi2, and IL-6) mRNA was not differentially expressed (*p* > 0.05) in either the caecal tonsil (Figure 9A) or spleen (Figure 9B) at 1–5d pi, when compared to uninfected controls. In contrast, *S*. Enteritidis infection stimulated higher levels of expression of chemokine mRNA, particularly in the caecal tonsil (Figure 9A). In the caecal tonsil, expression of CXCLi1, CXCLi2 and IL-6 was >2-fold higher on most experimental days and significantly greater (*p* < 0.05) at d2 pi (Figure 9A).

### 3.8. S. Gallinarum Suppresses Expression of Inflammatory Mediators in the Caecal Tonsil and Spleen and Promotes an Anti-Inflammatory IL-10 Response in the Spleen

**Figure 10:**
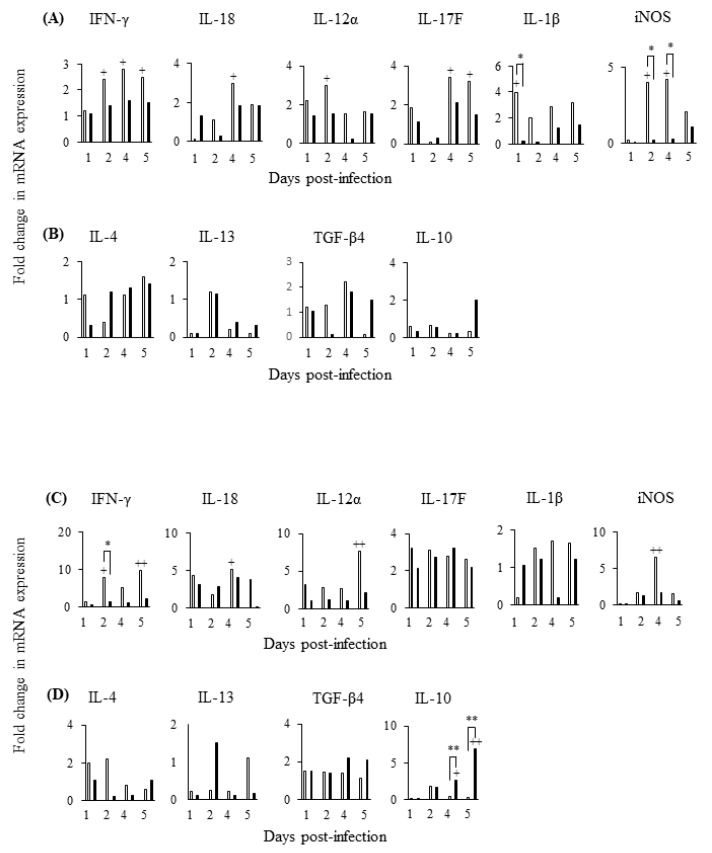
*S*. Gallinarum infection suppresses expression of pro-inflammatory cytokines and iNOS in both the caecal tonsils and spleen compared with *S*. Enteritidis but additionally increases anti-inflammatory IL-10 expression in the spleen. Mean expression of pro-inflammatory (**A**) and anti-inflammatory cytokine mRNA (**B**) is shown in the caecal tonsils and mean expression of pro-inflammatory (**C**) and anti-inflammatory cytokine mRNA (**D**) is shown in the spleen of chickens infected with *S*. Enteritidis (white bars) or *S*. Gallinarum (black bars) over a 5 day post-infection period. Means were determined from data obtained from 3 independent experiments (individual birds) and compared as fold changes to mRNA expression measured in uninfected controls (given an arbitrary value of 1). (+) indicates differences between levels of cytokines induced by each serovar compared to PBS-treated uninfected control, + *p* < 0.05, ++ *p* < 0.01; (*) indicates differences between levels of cytokines induced by different serovars, * *p* < 0.05, ** *p* < 0.01.

We then investigated whether a Th1 or Th2-like immune response predominated in secondary lymphoid organs (caecal tonsil and spleen) following experimental infection. In the caecal tonsil of *S*. Enteritidis-infected chickens, an inflammatory profile was evident throughout the infection period with significantly increased expression of IFN-γ mRNA at days 2, 4, and 5 pi, IL-12 (day 2), IL-18 (day 4) and IL-17F (days 4 and 5) compared to uninfected controls (Figure 10A). None of these cytokines were differentially expressed in the caecal tonsil of *S*. Gallinarum-infected chickens (*p* > 0.05) at any of the experimental time points investigated (Figure 10A). IL-1β expression was significantly greater (*p* < 0.05) in the caecal tonsil of *S*. Enteritidis-infected chickens, compared to *S*. Gallinarum-infected chickens, at d1 pi and expression of iNOS RNA was significantly greater on both d2 and d4 pi (*p* < 0.05) (Figure 10A). Expression of IL-4, IL-13, TGF-β4 or IL-10 mRNA was not significantly different (*p* > 0.05) in the caecal tonsil of chickens infected with either *S*. Enteritidis or *S*. Gallinarum compared to uninfected controls (Figure 10B).

An inflammatory profile was also evident in the spleens of *S*. Enteritidis-infected chickens compared to *S*. Gallinarum (Figure 10C). *S*. Enteritidis significantly increased expression of IFN-γ mRNA at days 2 and 5 pi (*p* < 0.05) compared to uninfected controls and on day 2 pi this was significantly greater (*p* < 0.05) than expression in *S*. Gallinarum-infected chickens. IL-12 (day 4), IL-18 (day 4), and iNOS (days 4) were also significantly increased in the spleens of *S*. Enteritidis-infected chickens compared to uninfected controls (Figure 10C). None of the inflammatory mediators investigated were differentially expressed in the spleens of chickens infected with *S*. Gallinarum, compared to uninfected controls (Figure 10C). *S*. Enteritidis infection did not significantly increase (*p* > 0.05) IL-4, IL-13, TGF-β4 or IL-10 mRNA expression in splenic tissues above levels measured in uninfected control. *S*. Gallinarum also did not significantly increase (*p* > 0.05) expression of IL-4, IL-13, TGF-β4 compared to uninfected controls. However, *S*. Gallinarum infection induced a significant increase (*p* > 0.01) in IL-10 mRNA expression in splenic tissues above that detected in either spleens from *S*. Enteritidis-infected chickens or uninfected control on both days 4 and 5 pi (*p* < 0.01) (Figure 10B).

## 4. Discussion

Our study shows that *S*. Enteritidis induces a dominant proinflammatory (Th1-like) immune response in chickens, in contrast to *S*. Gallinarum, which induces a decreased inflammatory response and more dominant anti-inflammatory, IL-10 response, in vivo. These differences in immune response may be the basis for the very different infection profiles of these serovars in chickens, with asymptomatic clearance of *S*. Enteritidis infection but the development of typhoid disease in chickens infected with *S*. Gallinarum.

It is appreciated that there may be limitations in the use of gene expression as a quantitative measure of cytokine production. However, when this work was initiated, good quality, reliable reagents for all the markers studied here were not available and it was considered better to use a single method rather than a combination of systems of quantification.

A previous study by Jones et al. [20] suggested that increased survival of *S*. Gallinarum in HD11 macrophages may be a platform for translocation from the intestine to deeper tissues. However, our study shows that *S*. Gallinarum did not survive as well as *S*. Enteritidis in chMDMs but the contradiction may be explained by our use of naturally derived macrophages, which probably better represent physiological populations of blood monocytes that migrate and differentiate into tissue macrophages during infection. A recent study using the *S*. Typhimurium/murine model of typhoid has also shown that *S*. Typhimurium is not carried within macrophages from the intestine and that although other cells such as dendritic cells and B lymphocytes may carry infection systemically, most carriage is done autonomously [21].

We show that expression of iNOS mRNA, NO_2_^−^ production, and expression of heterophil granulocyte chemoattractants CXCLi1, CXCLi2, and IL-6 are all decreased in *S*. Gallinarum-infected chMDMs in vitro and in the caecal tonsil and spleen in vivo, compared to *S*. Enteritidis infection. Production of reactive nitrogen species by innate immune cells during *Salmonella* infection is an important control mechanism [22], and reduced production of NO_2_^−^ has been reported in HD11 cells cultured with media aliquoted from *S*. Gallinarum-infected chick kidney cells (CKCs) compared to media aliquoted from *S*. Enteritidis-infected CKCs (2). We have previously shown that reduced NO_2_- production is a characteristic of typhoid versus non-typhoid *Salmonella* serovars in murine J774 macrophages [23]. Furthermore, reduced levels of CXCLi1, CXCLi2, and IL-6 expression may inhibit heterophil migration into infected tissues. Down-regulation of IL-6 has been reported in an epithelial cell model (chick kidney cells) infected with *S*. Gallinarum compared to *S*. Enteritidis [2]. In highly susceptible gnotobiotic piglets, early neutrophil recruitment prevents *Salmonella* transmission from the intestine and mortality [24] and the importance of heterophil recruitment in *Salmonella* resistance [25] and the role of CXCLi1 and 2 [26] has also been reported in chickens. Our study therefore shows reduced iNOS/NO_2_- and CXCLi1/CXCLi2 following *S*. Gallinarum infection correlates with low numbers in the caecum but rapidly increased colonisation of deeper tissues such as the liver. This, indicates that reduction in these inflammatory mediators may be the mechanism that facilitates transmission of typhoid serovars from the intestine to deeper tissues and failure to reduce these mediators prevents transmission from occurring during infection with non-typhoid serovars.

In vitro studies, measuring output from CD4^+^ lymphocytes cultured with infected chMDMs, showed that both serovars induced greater expression of co-stimulatory markers (CD40, CD80, and CD86), phasic expression of lymphocyte activation or deactivation markers (CD28 and CTLA-4 respectively) and increased CD4^+^ lymphocyte proliferation, compared to controls. Although both serovars stimulated significant expression of IL-10 in chMDMs, these did not induce development of IL-10 or TGF-β expressing CD4^+^ lymphocytes in co-cultures. *S*. Gallinarum strain 9 and *S*. Enteritidis strain P125109 (phage type 4) used in this study have been shown previously to be representative of field strains [27,28] by producing typical disease [29]. However, we also repeated the chMDM infections using a wider selection of *S*. Enteritidis strains, including an additional 2 different phage types, isolated from cases of human food poisoning due to poultry consumption, and an additional three strains of *S*. Gallinarum isolated from cases of fowl typhoid. Although we show some strain variation, the overall effect of the two serovars were the same, which suggested that the two type strains were good models of infection with each serovar. Therefore, CD4^+^ lymphocytes cultured with *S*. Gallinarum-infected chMDMs also did not produce significant levels of IL-10, TGF-β, or IL-4/IL-13, thus indicating that *S*. Gallinarum did not induce a tolerogenic or a Th2 phenotype. This is in contrast to *S*. Pullorum infection in chickens, which also transits from the intestine to deeper organs but causes a switch from a resolving Th1 phenotype (seen in *S*. Enteritidis infection) to a non-resolving Th2 phenotype and subsequent carrier status [18].

Another striking difference in the immune profile of these serovars was a dominant *S*. Enteritidis-induced Th1-like response with high levels of IFN-γ and IL-17 and very low levels of IL-10, IL-4, IL-13 and TGF-β in the caecal tonsil and spleen in vivo. In comparison, *S*. Gallinarum infection induced much lower levels of IFN-γ and IL-17 in vitro, with no differential expression of these cytokines in the caecal tonsil and much lower levels in the spleen.

However, as well as reducing inflammatory mediators, *S*. Gallinarum infection significantly increased expression of IL-10 in the spleen; which did not occur during *S*. Enteritidis infection. Reduction in expression of pro-inflammatory cytokines and increased expression of anti-inflammatory IL-10 may provide a less hostile environment within deeper organs, which facilitates *S*. Gallinarum growth or at least maintenance of the bacterial populations within these organs. Similarly, decreased levels of inflammatory mediators, including IFN-γ and IL-17 [10] and TNF-α and IL-6 [30] have been reported in the serum of humans with acute typhoid. These immune mediators were increased during convalescence, thus indicating their importance in the development of typhoid (reduced by infection) and their role in immune resolution (upregulation). A study by Jansen et al. [13] indicated that inhibition of inflammatory immune mediators (TNF-α and CXCL2) by *S*. Typhi occurs via the Vi capsular antigen. Using a chimeric Vi antigen insert into *S*. Typhimurium, this latter study showed that infection in mice not only inhibited TNF-α and CXCL2 but also increased anti-inflammatory IL-10 production. The fact that wild type *S*. Typhimurium were unable to do so may suggest that the *S*. Gallinarum/chicken model rather than the *S*. Typhimurium/murine model better reflects *S*. Typhi infection in humans.

In conclusion, our study shows a differential immune response induced by typhoid and non-typhoid *Salmonella*, which may explain the mechanism behind the evolution of typhoid disease. We hypothesise that *S*. Gallinarum (unlike *S*. Enteritidis) reduces expression of inflammatory mediators in intestinal tissue, which allows for transmission to deeper organs. We also hypothesise that *S*. Gallinarum not only reduces inflammation within these organs but also stimulates an anti-inflammatory IL-10 response, which then establishes and maintains the infection within these organs. Thus, these findings indicate that *S*. Gallinarum infection in chickens has potential as an alternative model of human typhoid for future study.
